# Foot Trajectory Features in Gait of Parkinson’s Disease Patients

**DOI:** 10.3389/fphys.2022.726677

**Published:** 2022-05-04

**Authors:** Taiki Ogata, Hironori Hashiguchi, Koyu Hori, Yuki Hirobe, Yumi Ono, Hiroyuki Sawada, Akira Inaba, Satoshi Orimo, Yoshihiro Miyake

**Affiliations:** ^1^ Department of Computer Science, Tokyo Institute of Technology, Yokohama, Japan; ^2^ Department of Computational Intelligence and System Science, Tokyo Institute of Technology, Yokohama, Japan; ^3^ Department of Neurology, Kanto Central Hospital, Tokyo, Japan

**Keywords:** foot trajectory, gait, inertial measurement unit, Parkinson’s disease, forward displacement, vertical displacement

## Abstract

Parkinson’s disease (PD) is a progressive neurological disorder characterized by movement disorders, such as gait instability. This study investigated whether certain spatial features of foot trajectory are characteristic of patients with PD. The foot trajectory of patients with mild and advanced PD in on-state and healthy older and young individuals was estimated from acceleration and angular velocity measured by inertial measurement units placed on the subject’s shanks, just above the ankles. We selected six spatial variables in the foot trajectory: forward and vertical displacements from heel strike to toe-off, maximum clearance, and change in supporting leg (F1 to F3 and V1 to V3, respectively). Healthy young individuals had the greatest F2 and F3 values, followed by healthy older individuals, and then mild PD patients. Conversely, the vertical displacements of mild PD patients were larger than the healthy older individuals. Still, those of healthy older individuals were smaller than the healthy young individuals except for V3. All six displacements of the advanced PD patients were smaller than the mild PD patients. To investigate features in foot trajectories in detail, a principal components analysis and soft-margin kernel support vector machine was used in machine learning. The accuracy in distinguishing between mild PD patients and healthy older individuals and between mild and advanced PD patients was 96.3 and 84.2%, respectively. The vertical and forward displacements in the foot trajectory was the main contributor. These results reveal that large vertical displacements and small forward ones characterize mild and advanced PD patients, respectively.

## 1 Introduction

Parkinson’s disease (PD) is a complex neurological disorder in which the dopaminergic neurons of the substantia nigra gradually degenerate (Post et al., 2007; Jankovic 2008; Haji Ghassemi et al., 2018). Bradykinesia, akinesia, muscular rigidity, and resting tremor are typical movement-related symptoms of PD (Fahn 2008). These symptoms affect gait instability in PD patients, which is known to progress with severity (Jankovic et al., 2000; Hausdorff 2009; Hass et al., 2012). The gait instability decreases patients’ independence and quality of life. Thus, it is necessary to understand the characteristics of PD patients’ gait for the proper evaluation and treatment of gait alternation (Mirelman et al., 2019).

Spatiotemporal gait features measured in subjects through motion measurement technology, such as pressure sensors, optical motion capture, and wearable sensors, have been successfully used to evaluate the gait of PD patients (Mirelman et al., 2019). Wearable sensors can be used for diagnosis and monitoring in both clinical and daily living settings, and are useful because of their relatively low cost and usability, as compared to non-wearable solutions, such as optical motion capture systems, and force platforms, which are the current gold standard for motion analysis (de Oliveira Gondim et al., 2020). In particular, inertial measurement units (IMUs), which consist of accelerometers, gyroscopes, and magnetometers, can be used to determine spatiotemporal and frequency features of the gait, such as stride and step-lengths, as well as swing/stance/stride duration, from acceleration and/or angular velocity (Chang et al., 2016; Del Din et al., 2016; Micó-Amigo et al., 2019).

These measurement technologies have revealed that spatial–temporal gait parameters are altered in PD patients as compared to healthy individuals. Gait velocity becomes slower and stride/step-length is shorter than in age-matched healthy controls, and these symptoms worsen with disease progression (Alban et al., 2014; Galna et al., 2015; Pistacchi et al., 2017; Mirelman et al., 2019). PD patients compensate for this short stride/step-length by increasing cadence which can cause suffering gait (Morris et al., 1994a; Morris et al. 1994b; Morris et al. 1996). The rhythmic pattern of gait in PD patients also changes with progression (Hass et al., 2012; Djurić-Jovičić et al., 2017; Micó-Amigo et al., 2019). For instance, the inter-limb asymmetry in gait, such as step duration, is well-known in the early stages of PD (Djurić-Jovičić et al., 2017). In patients with mild PD, the ratio of the swing/stance duration is increased and decreased, respectively (Hass et al., 2012). Variability in gait parameters, such as step duration, is larger in PD patients than in age-matched older individuals (Rochester et al., 2014).

Compared to the temporal and frequency features, little is known about the spatial features of PD patients’ gait, such as foot trajectory. This may be because evaluation of the foot trajectory requires detailed measurements of spatial features for optical motion capture, which involves high cost and places a heavy load on the participants. Recently, developments in the analysis of IMU data have improved the accuracy of the estimation of the foot trajectory during gait (Bamberg et al., 2008; Mariani et al., 2012; Kitagawa and Ogihara 2016; Kluge et al., 2017; Tunca et al., 2017; Hori et al., 2020; Mao et al., 2021). For instance, Hori et al. (2020) accurately estimated stride-by-stride foot trajectories using IMUs attached to the left and right shanks, just above the ankles. The stride-lengths estimated using this method and those from an optical motion capture system were correlated, obtaining a coefficient of determination of approximately 0.98.

The state-of-the-art technology involved in wearable IMUs could reveal more detailed spatial features of the gait of PD patients. This study investigated foot trajectory features in PD patients and their differences from healthy older and young individuals. The temporal and frequency gait features of patients with mild PD are not markedly different from those of healthy older individuals, compared to the difference between advanced PD patients and healthy older individuals (Klucken et al., 2013; Rovini et al., 2017). Thus, investigating gait trajectory features in patients would be useful for understanding the gait of mild PD patients. We recruited healthy young individuals to separate the effects of aging and PD on the foot trajectory as older people are susceptible to developing PD. To investigate the effects of the severity of PD on foot trajectory, we also recruited advanced PD patients. For these purposes, we measured the foot trajectory of subjects using IMUs attached to both shanks. In addition, to determine whether the foot trajectory captures the gait characteristics of PD patients, we used principal component analysis and distinguished PD patients from healthy controls using machine learning technology.

## 2 Methods

### 2.1 Participants

Thirty mild PD patients (modified Hoehn and Yahr [H&Y] scale stages 1–2; age, 68.4 ± 10.3 years; 16 women and 14 men), 24 healthy older individuals (age, 70.7 ± 3.9 years; 12 women and 12 men), 34 healthy young individuals (age, 24.6 ± 3.5 years; 10 women and 24 men), and 27 advanced PD patients (modified H&Y scale at stages 2.5–4; age, 77.3 ± 5.3 years; 15 women and 12 men) participated in this study. All PD patients were being treated with anti-Parkinsonian drugs at the time of the study. During the experiment, patients were in the on-state, in which they responded positively to the medication. We excluded patients with PD who exhibited freezing of gait and those who needed walking assistance during the experiment. None of the participants suffered from any musculoskeletal or neurological pathologies other than PD. The study was conducted in accordance with the Declaration of Helsinki and was approved by the Research Ethics Review Committee of the Tokyo Institute of Technology and the Ethics Committee of Kanto Central Hospital. Written informed consent was obtained from all participants.

### 2.2 Experiment Task

**FIGURE 1:**
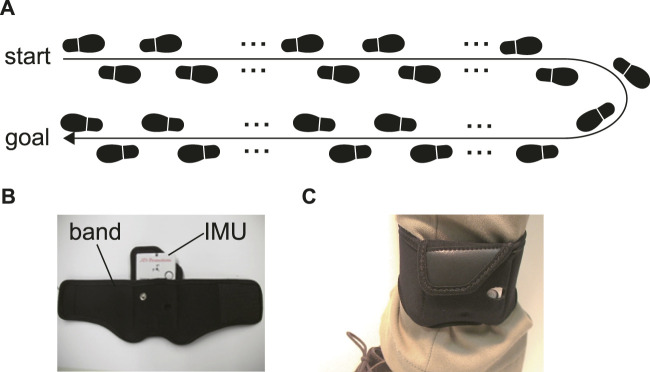
**(A)** The experiment task. Participants walked and turned in a straight corridor, giving over 60 strides. **(B)** The IMU and a belt to attach the IMU on the shank. **(C)** The belt with the IMU around the shank.

We asked each participant to walk back and forth in a straight corridor, giving over 60 strides (Figure 1A). The participants walked alone without any type of walking support. The initial and final five strides and those corresponding to direction change were excluded from the data analysis. We used 40 strides in total (20 strides on the left and 20 on the right).

### 2.3 Foot Trajectory Estimation

The acceleration and angular velocity of the subjects’ shanks for foot trajectory estimation were measured using IMUs (TSDN121 ATR-Promotions, Seika, Japan) (Figure 1B). The ranges of the triaxial accelerometer and gyroscope were ±8 G and ±1,000 dps, respectively, and the sampling frequency was 100 Hz. The IMU dimensions were 37 × 46 × 12 mm and the device weighed 22 g. This sensor was attached to the shank by means of a band, just above the ankle (Figures 1B, C).

We used our previous method for foot trajectory estimation (Hori et al., 2020), which was conducted stride-by-stride, to reduce accumulated error. Specifically, the time series of accelerations and angular velocities were divided at the time when the shank movement was the steadiest, corresponding to the lowest pitch velocity of the shank. This timing was set as the initial timing of each stride during the heel strike. The heel strike was measured using angular velocity in the sagittal plane because this value changes on the spike with a heel strike. The timing of the spike was set the timing of the heel strike. The acceleration vector was transformed from the sensor coordinate system to a world coordinate system based on the assumption that the accelerometer only detects gravity at mid-stance in the gait cycle because the shank where the IMU was attached hardly move at this time. The initial angular velocity was estimated using the gravity vector of the accelerometer. The foot trajectory was then calculated as the double integral of the acceleration vector. The effect of drift due to IMU error, we performed forward and backward integration of the foot acceleration and used weighted averages as foot velocity, assuming that the initial, and final velocities in the three directions and the position along the vertical direction were zero.

### 2.4 Statistical Method

#### 2.4.1 Features in Foot Trajectory

**FIGURE 2:**
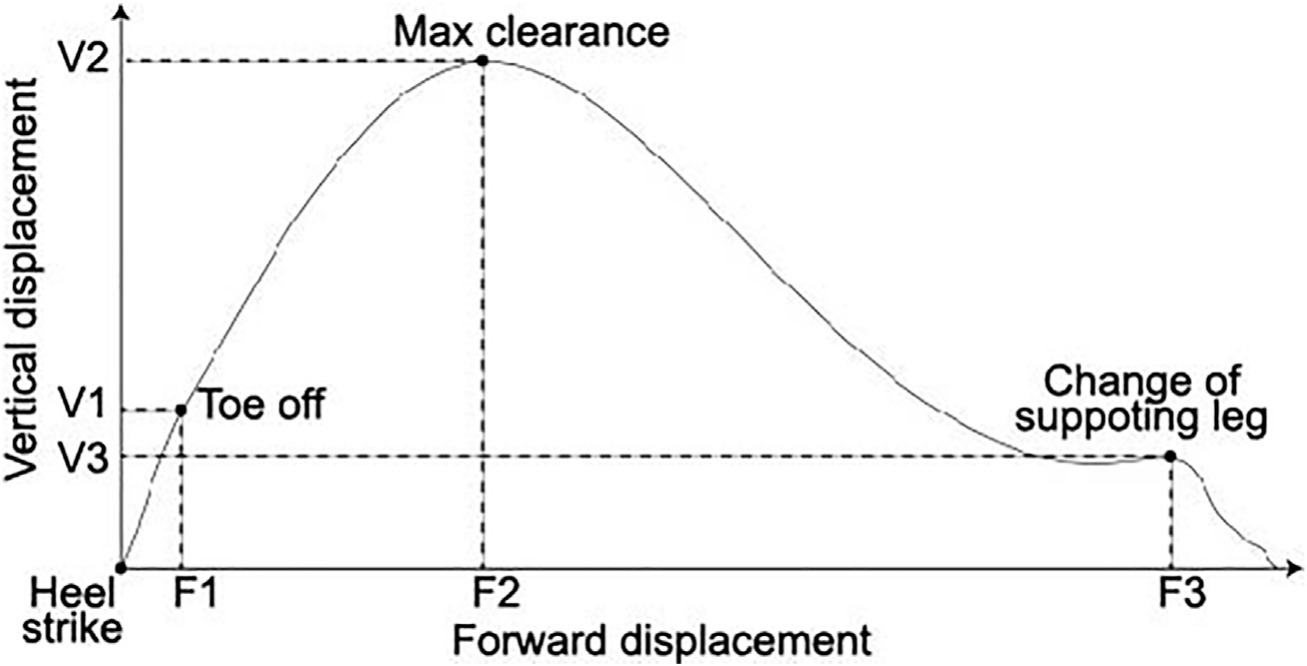
Foot trajectory and six spatial features of the foot trajectory: forward and vertical displacements from the heel strike to toe-off (F1 and F2, respectively), maximum clearance (F2 and V2, respectively), and change of supporting leg (F3 and V3, respectively). The vertical and horizontal axes are the vertical and forward displacements, respectively.

For the foot trajectory features, we used three forward and three vertical displacements, as shown in Figure 2: forward and vertical displacements from the onset of the stride (heel strike) to toe-off (F1 and V1, respectively), the timing when the foot reached the highest point (maximum clearance) (F2 and V2, respectively), and the timing when the supporting leg changed (F3 and V3, respectively).

For the statistical test, we used a one-way ANOVA. All post hoc tests were conducted using the Shaffer Method. The significance level was set at 0.05 for all tests. In addition, we conducted principal component analysis to investigate the trajectory features in detail. We determined the number of a principal components using a parallel analysis. These analyses were conducted using R ver. 3.2.4.

#### 2.4.2 Distinguishing Method

For distinguishing the PD and healthy groups, we used a supervised learning algorithm: the soft-margin kernel support vector machine (SVM). Some studies showed better classification accuracy between PD patients and healthy older individuals and/or between patients with different PD severities using the SVM than when using other classification methods (Klucken et al., 2013; Caramia et al., 2018). We conducted binary distinguishing between mild PD patients and healthy older individuals, mild, and advanced PD patients, mild PD patients and healthy young individuals, and healthy older and healthy young individuals. Because PD is a progressive disorder, distinguishing between patients with advanced PD and healthy individuals was not considered important. We used 10-fold cross-validation. First, the dataset was divided into 10 balanced groups. Then, one group was used for the test and the rest for training. Testing and training were repeated until all the groups were considered for validation. The participant data were included in both the training and test datasets. Finally, accuracy was defined as the average accuracy for 10-fold cross-validation. For SVM analysis and cross-validation, we used MATLAB statistics and a machine learning toolbox (MathWorks, Inc., Natick, MA, United States).

## 3 Results

### 3.1 Averages of the Forward and Vertical Displacements

**FIGURE 3:**
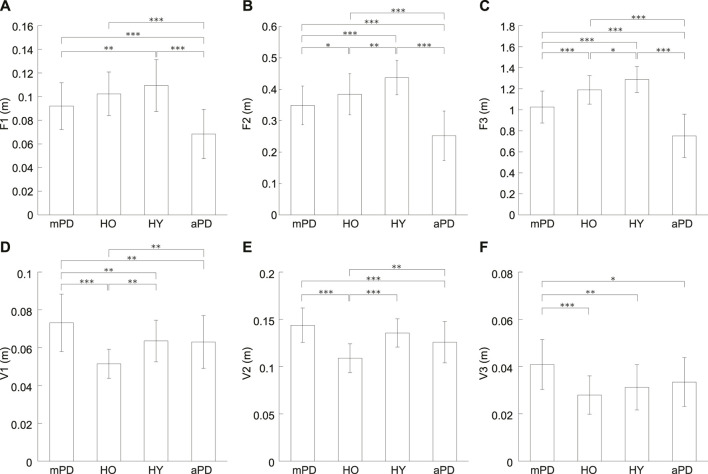
Averages of the forward and vertical displacements. **(A)** F1, **(B)** F2, **(C)** F3, **(D)** V1, **(E)** V2, and **(F)** V3. mPD, HO, HY, and aPD represent mild PD patients, healthy older individuals, healthy young individuals, and advanced PD patients, respectively. The error bars indicate the standard deviations between the participants. *, **, and *** represent *p* < 0.05, *p* < 0.01, and *p* < 0.001, respectively.

Figure 3 shows averages of the forward and vertical displacements for each group. In the forward displacements (Figures 3A–C), the average for the healthy young individuals seems to be the largest, followed in order by the healthy older individuals, mild PD patients, and advanced PD patients. A one-way ANOVA showed significant differences for all of the forward displacements (*p* < 0.001 in all).

Multiple comparisons testing showed that there was no significant difference for F1 between the mild PD patients and healthy older individuals (*p* = 0.129) F1 of mild PD patients was significantly smaller than the healthy young individuals (*p* = 0.003). In contrast, there was no significant difference in F1 between healthy older and young individuals (*p* = 0.200). The F1 of the advanced PD patients was smaller than the mild PD patients, healthy older individuals, and healthy young individuals (all *p* < 0.001).

However, the results of a multiple comparisons test showed there were significant differences for F2 between all groups. F2 of mild PD patients was smaller than the healthy older individuals (*p* = 0.048). Furthermore, F2 of mild PD patients and healthy older individuals were smaller than the healthy young individuals (*p* < 0.001 and *p* = 0.006, respectively). F2 of the advanced PD patients was smaller than the other three groups (all *p* < 0.001).

Multiple comparisons also showed significant differences for F3 between all groups. F3 of mild PD patients was smaller than healthy older individuals (*p* < 0.001). In addition, F3 of mild PD patients and healthy older individuals was smaller than healthy young individuals (*p* < 0.001 and *p* = 0.019, respectively). F3 of the advanced PD patients was smaller than the other groups (all *p* < 0.001).

In the vertical displacements (Figures 3D–F), the average of the mild PD patients was the largest and that of the healthy older individuals seems to be the smallest. For all of the vertical displacements (V1–V3), a one-way ANOVA revealed significant differences (*p* < 0.001 in all).

Multiple comparisons showed that V1 of the mild PD patients was significantly larger than the healthy older individuals (*p* < 0.001). V1 of the mild PD patients was also significantly larger than the healthy young individuals (*p* = 0.001). Conversely, the V1 of healthy older individuals was significantly smaller than healthy young individuals (*p* = 0.001). The advanced PD patients were significantly smaller for V1 than mild PD patients (*p* = 0.008). In addition, V1 of the advanced PD patients was significantly smaller than the healthy older individuals (*p* = 0.004) but not healthy young individuals (*p* = 0.877).

The multiple comparison revealed that V2 of mild PD patients was significantly larger than the healthy older individuals (*p* < 0.001). There was no significant difference for V2 between mild PD patients and healthy young individuals (*p* = 0.070). In contrast, V2 of healthy older individuals was significantly smaller than healthy young individuals (*p* < 0.001). V2 of the advanced PD patients was significantly smaller than mild PD patients and healthy older individuals (*p* < 0.001 and *p* = 0.003, respectively) but not than healthy young individuals (*p* = 0.070).

V3 of mild PD patients was significantly larger than the healthy older individuals (*p* < 0.001). V3 of the mild PD patients was also significantly larger than young individuals (*p* < 0.001). Oppositely, there was no significant difference between healthy older and young individuals (*p* = 0.426). V3 of the advanced PD patients was significantly smaller than the mild PD patients (*p* = 0.015). There was no significant difference for V3 of advanced PD patients from the healthy older individuals and healthy young individuals (*p* = 0.137 and *p* = 0.426, respectively).

### 3.2 Principal Component Analysis

**FIGURE 4:**
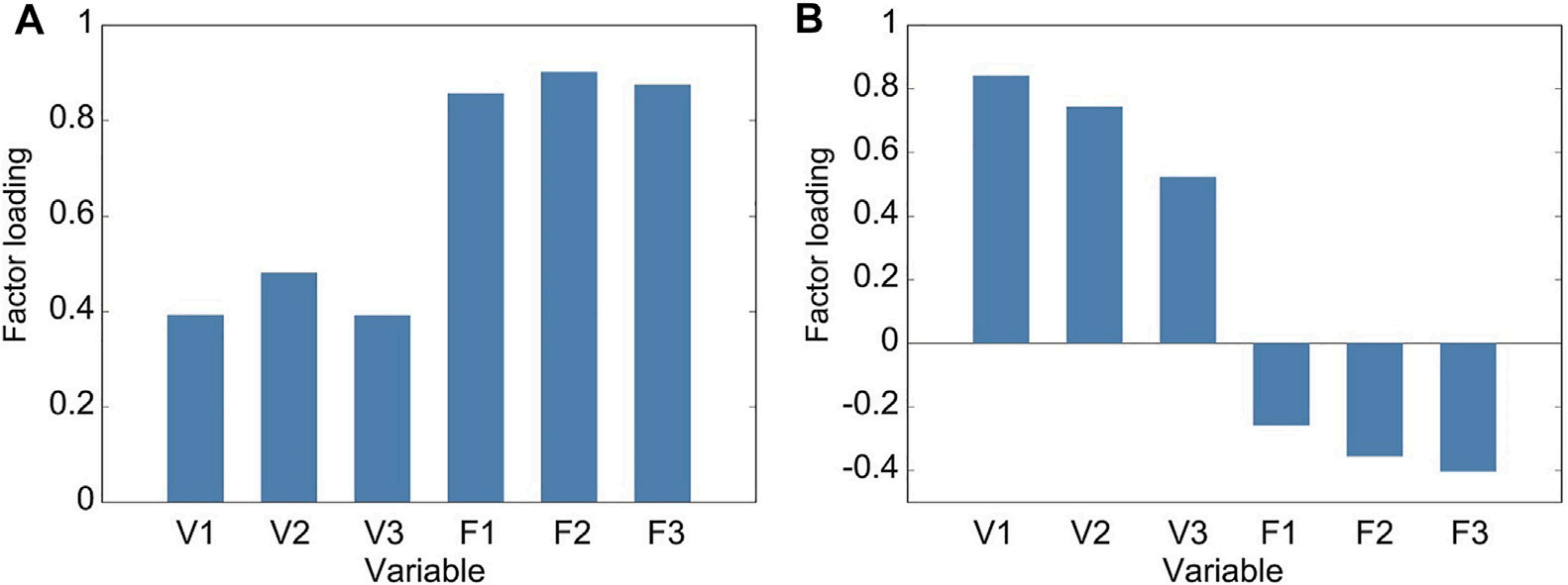
Factor loadings of principal components from gait spatial features. Loading is the positive or negative correlation between a principal component and a variable. **(A)** Factor loadings of the first principal component, which is strongly and positively correlated to variables in the forward displacement (F1 to F3) and hence describes this displacement in the foot trajectory. **(B)** Factor loadings of the second principal component, which is strongly and positively correlated to variables in the vertical displacement (V1 to V3) and hence describes this displacement in the foot trajectory.

The averages in the forward and vertical displacements showed similar tendencies, respectively. To investigate this tendency, we conducted the principal component analysis. Table 1 lists the standard deviations, variance proportions, and their accumulated value for the principal components. The cumulative proportion is the total variation in the features explained up to a particular principal component. The parallel analysis suggested two principal components. Figure 4 shows the factor loadings for the first and second principal components, corresponding to the correlation between the components and variables. The first principal component showed a high correlation with the variables along the forward displacement, F1–F3 (Figure 4A). Larger variable values induced positively larger loadings of the first principal component. The second principal component showed a high correlation with the variables along the vertical displacement, V1–V3 (Figure 4B). Larger variable values induced positively larger loadings of this component. Therefore, the first and second components mainly represent the displacements in the foot trajectory along the forward and vertical displacements, respectively.

**TABLE 1:** Standard deviation and variance proportion of principal components for PCA feature extraction.

Principal component	1	2	3	4	5	6
Standard deviation	1.690	1.375	0.853	0.633	0.316	0.162
Variance proportion	0.476	0.315	0.121	0.067	0.016	0.004
Cumulative proportion	0.476	0.791	0.912	0.979	0.996	1.000

**FIGURE 5:**
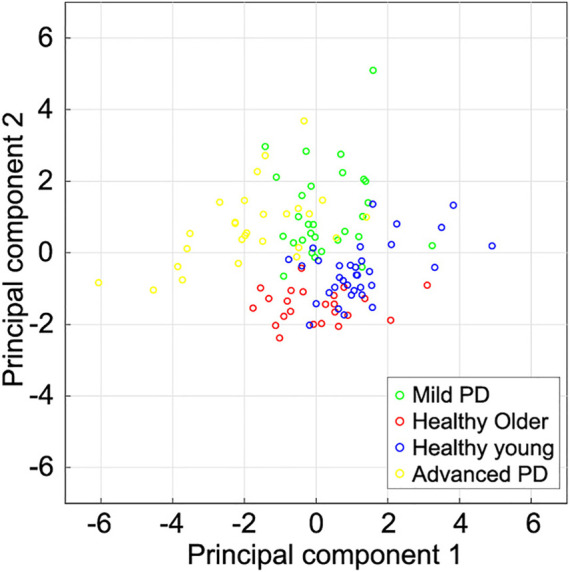
Scatter plot of the first two principal components extracted from subjects’ spatial gait features. The first and second component magnitudes represent the displacements of the foot trajectories in the forward and vertical directions, respectively. Healthy young individuals tended to have a larger first principal component than other subjects. The first principal component of mild PD patients and healthy older individuals is larger than that of advanced PD patients. The second principal component of healthy older individuals tends to be smaller than that of the other subjects.

Figure 5 shows the scatter plot of the first and second principal components from the subjects in this study. Although there were significant differences in F2 and F3 between healthy older individuals and mild PD patients, the first principal component, which mainly related to forward displacements, tended to be similar. The second principal component, mainly related to vertical displacements, of the mild PD patients seems smaller than healthy older individuals. Comparing healthy older and young individuals, both components tended to be smaller in older individuals than in young individuals. In contrast, the first principal component in mild PD patients also tended to be smaller than in young individuals, but their second principal component tended to be larger. The first principal component of many advanced PD patients was smaller than patients with mild PD.

### 3.3 Distinguishing of PD Patients

To investigate the tendency shown in the principal component analysis, we conducted a soft-margin kernel SVM to binary distinguish each category of subjects, considering four pairs of subjects: mild PD patients and healthy older individuals, mild PD, and advanced PD patients, mild PD patients and, healthy young individuals, and healthy older and healthy young individuals. We did not distinguish the advanced PD patients from healthy individuals because differences in gait are more obvious than between mild PD patients and healthy individuals (Klucken et al., 2013; Rovini et al., 2017).

**FIGURE 6:**
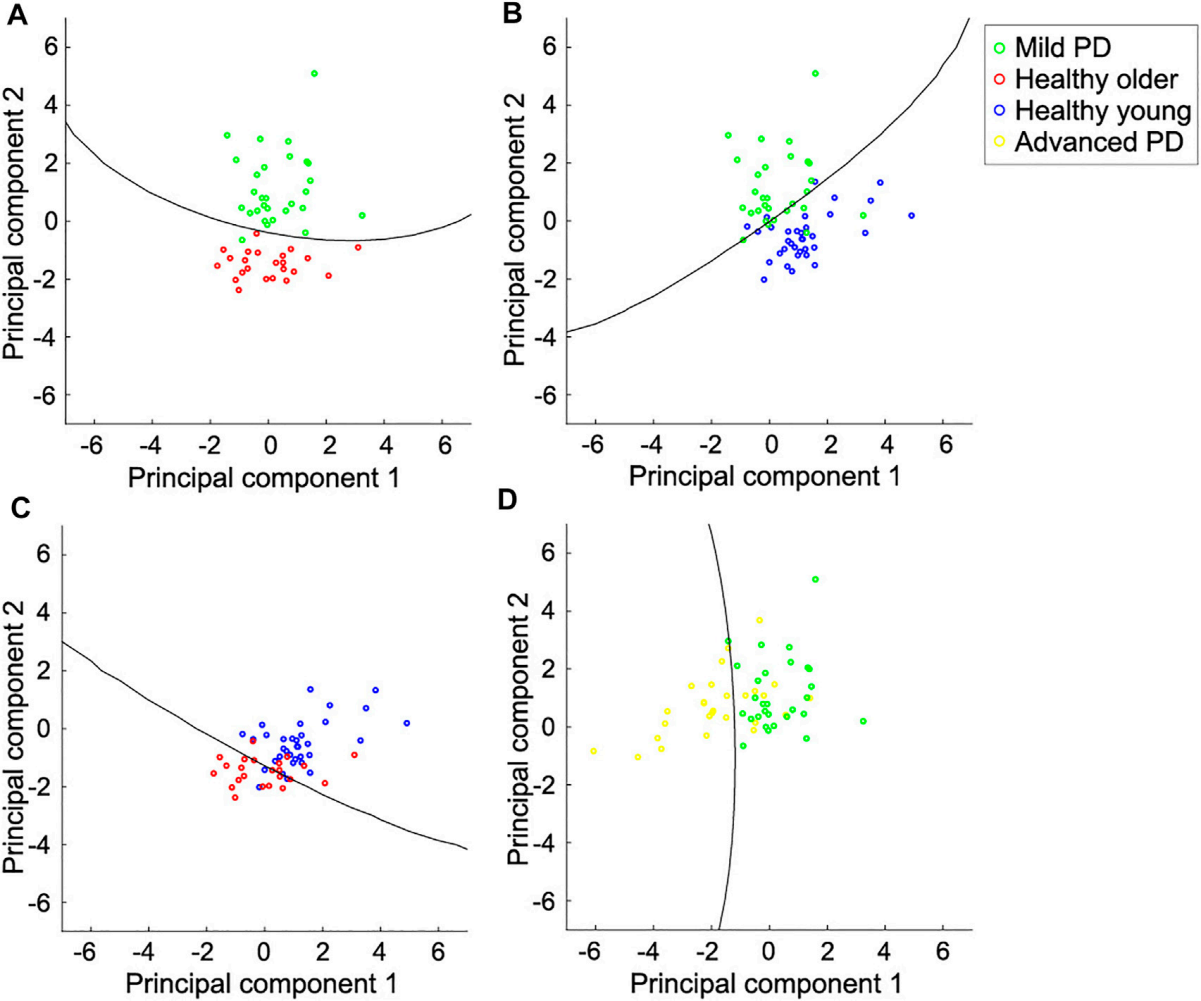
Scatter plot for pairs of individual categories and boundaries using the first two principal components. **(A)** Mild PD patients and healthy older individuals. They are mainly distinguished by the second principal component, which represents the vertical displacements in the foot trajectory. **(B)** Mild PD patients and healthy young individuals. They are distinguished by both components. The foot trajectory of the mild PD patients tended to be larger in the vertical direction and smaller in the forward direction than in healthy young individuals. **(C)** Healthy older individuals and young individuals. They are also distinguished by both components. The foot trajectory of the healthy older individuals tended to be shorter in both directions than in healthy young individuals. **(D)** Mild and advanced PD patients. They are mainly distinguished by the first principal component, which represents the forward displacements. Each circle represents one subject, and the lines represent the boundaries between the two categories.

**FIGURE 7:**
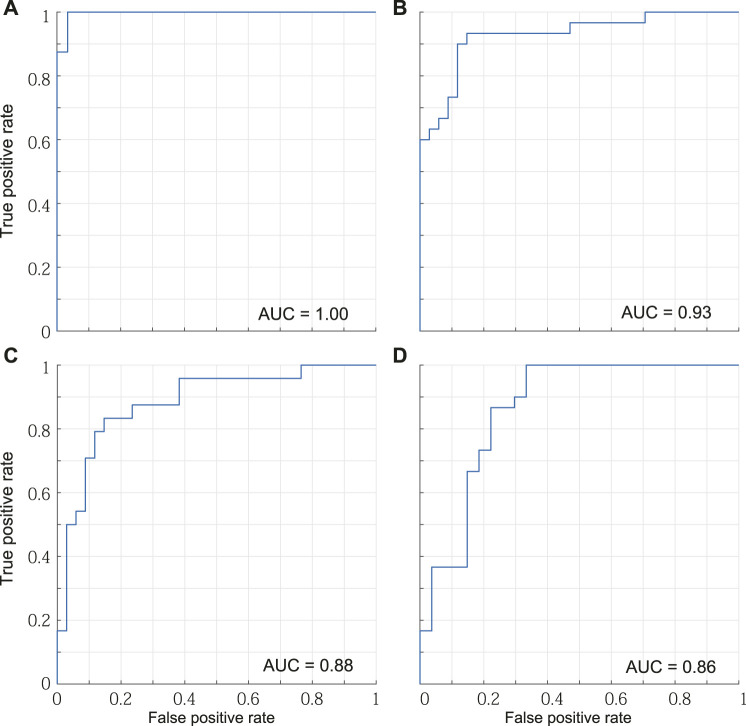
ROC curves and AUCs. **(A)** Mild PD patients and healthy older individuals. **(B)** Mild PD patients and healthy young individuals. **(C)** Healthy older individuals and young individuals. **(D)** Mild and advanced PD patients.

Table 2 lists the accuracy of distinction using the six spatial variables and the first two principal components. The accuracy of distinguishing between mild PD patients and healthy older individuals was the highest among all pairs, reaching 96.3% when using either all the variables or the first two principal components. Although the PCA did not lead to better results over using all variables, the characteristics of the displacements in the trajectory were well contracted by the PCA. Figure 6 shows the boundaries between the groups obtained by using the first two principal components, and Figure 7 shows the ROC curves and AUCs. The boundary curve between patients with mild PD and healthy older individuals (Figure 6A) is virtually horizontal. Hence, mild PD patients and healthy older individuals were mainly separated by the second principal component, which represents the vertical displacements (V1, V2, and V3). To investigate whether all three vertical displacements or second component contributed to the accuracy of distinguishing between mild PD patients and healthy older individuals, we distinguished them using only V1, V2, or V3 with the same SVM. The accuracies of the vertical variables V1, V2, and V3 were 79.6, 83.3, and 74.1%, respectively.

**TABLE 2:** Distinguishing accuracy (in percentages).

Pair of subjects	Features	
	All variables	First two principal components
Mild PD patients–Healthy older individuals	96.3	96.3
Mild PD patients–Healthy young individuals	92.7	89.1
Healthy older individuals—Healthy young individuals	77.6	82.8
Mild PD–Advanced PD patients	82.5	84.2

The other accuracies varied between 77.6 and 92.7% for all the variables, and between 82.8 and 89.1% for the first two principal components. Hence, the categories could be suitably distinguished using the first two principal components, which represent the forward and vertical displacements, respectively. Mild PD patients and healthy young individuals were distinguished by both of the first two principal components (Figure 6B). The first principal component of patients with mild PD was smaller, while the second principal component was larger, than that of healthy young individuals. Hence, the foot trajectory of patients with mild PD exhibited shorter forward and larger vertical displacements than that of healthy young individuals. Healthy older individuals and young individuals were also distinguished by both the first and second principal components (Figure 6C). In this case, healthy older individuals exhibited smaller first and second principal components than healthy young individuals, suggesting that the foot trajectory of the former category tends to be shorter in both the forward and vertical directions than that of the latter category by the effects of aging. Patients with mild or advanced PD were mainly distinguished by the first principal component, which represents the forward displacements (F1, F2, and F3) (Figure 6D).

We investigated how accurately each category was predicted using confusion matrices obtained using the principal component method, as shown in Table 3. Both mild PD patients and healthy older individuals were accurately predicted, with 96.7 and 95.8% accuracy, respectively. In contrast, advanced PD patients were poorly distinguished from mild PD patients, with an accuracy reaching only 67.7%.

**TABLE 3:** Confusion matrix for distinguishing subject categories using the first two principal components (in percentages).

		Obtained				Obtained	
		mPD	HO			mPD	HY
**True**	**mPD**	96.7	3.3	True	mPD	87.0	13.3
	**HO**	4.2	95.8		HY	15.0	85.0

		**Obtained**				**Obtained**	
		**HO**	**HY**			**mPD**	**aPD**
**True**	**HO**	71.0	29.0	True	mPD	100	0
	**HY**	8.8	91.2		aPD	33.3	67.7

Percentages along the diagonal and off-diagonal represent the correct and incorrect prediction rates, respectively. mPD, HO, HY, and aPD represent mild PD patients, healthy older individuals, healthy young individuals, and advanced PD patients, respectively.

## 4 Discussion

This study aimed to investigate the characteristics of PD patients’ foot trajectories during gait, which was estimated using wearable sensor data. First, we investigated the difference in foot trajectory between mild PD patients and healthy older individuals because the temporal and frequency gait features of these groups are not very different (Klucken et al., 2013; Rovini et al., 2017). Secondly, to differentiate between the effects of aging and PD on foot trajectory, we investigated the foot-trajectory difference between mild PD patients as well as healthy older individuals and healthy young individuals. Finally, to investigate the effect of the severity of PD on foot trajectory, we compared the foot trajectories of mild and advanced PD patients. We used six spatial features of the foot trajectory: the forward and vertical displacements from heel strike to toe-off, maximum clearance, and change of supporting leg.

Although there was no significant difference for F1 between the mild PD patients and healthy older individuals, F2 and F3 of the mild PD patients was significantly smaller than the healthy older individuals In contrast, the mild PD patients’ all vertical displacements (V1–V3) were significantly larger than the healthy older individuals. Thus, compared to the healthy older individuals, the trajectory of the mild PD patients was characterized as a small forward displacement except for toe-off and large vertical displacement.

In addition, compared to the healthy young individuals, the forward displacement of the older individuals was smaller and that of the mild PD patients even smaller. However, there was no significant difference for F1 between the healthy older and young individuals. Thus, the forward displacements of the healthy the mild PD patients are considered small because of both aging and PD. On the other hand, the all vertical displacements of the mild PD patients were larger than the healthy young individuals. This is in contrast to the smaller V1 and V2 of the healthy older individuals compared to the healthy young individuals. Thus, the mild PD patients raised their foot more than the decreasing of the vertical displacements due to the effects of aging.

The contrast shown in the vertical displacements between the mild PD patients and healthy older individuals could characterize the gait of the mild PD patients more than the forward displacements. This was supported by the results of the principal component analysis and distinguishing using the SVM. The first and second principal components obtained by PCA were mainly related to the displacements in the forward and vertical directions, respectively. The accuracy for distinguishing mild PD patients from healthy older individuals using the first two components reached 96.3%. These two categories were mainly distinguished by the second component (Figure 6A), which is related to the vertical displacements in the foot trajectory.

Regarding the distinction between healthy older and healthy young individuals, the vertical displacements of older individuals tended to be shorter than that of the young individuals (Figure 6D). Nagano et al. found that max clearance of toe of healthy older individuals was lower than that of healthy young individuals (Nagano et al., 2011). Our results showed that healthy older individuals raise their toe and lower shank lower than healthy older individuals. The vertical displacements of patients with mild PD was greater than that in healthy young individuals (Figure 6C). These results suggested that foot clearance in patients with mild PD was larger than in healthy older and healthy young individuals. Although foot clearance during gait in PD patients at stages 1–3 of the H&Y scale has been reported to be smaller than that in healthy young individuals (Alcock et al., 2016), foot clearance in patients with mild PD (i.e., those with H&Y scale in stages 1 and 2) remains unclear. Foot clearance in faller older individuals is larger than that in non-faller older individuals (Barrett et al., 2010). Thus, mild PD patients lift their feet considerably to avoid falling, which is a common concern for patients with PD.

The accuracy in distinguishing mild PD patients and healthy older individuals when using each vertical displacement (V1–V3) was lower than when using all variables and when using the first two principal components. This finding suggests that the foot clearance of patients with mild PD did not increase uniformly compared to healthy older individuals. In fact, the factor loadings in the second component were different among V1, V2, and V3 (see Figure 4B). This reflects the gait trajectory feature in PD patients. The gait of PD patients is less automatic and requires more cognitive resources. For this reason, dual tasks impair PD patients, causing falls (Rochester et al., 2014). In addition, prefrontal activation increases in PD patients during imaginary gait tasks, which reflects the increase in cognitive demand for gait (Peterson et al., 2014; Maidan et al., 2016). Thus, our results reflect the less automatic and more intentional gait of PD patients.

All forward and vertical displacements of advanced PD patients were smaller than mild PD patients. This result suggests that the whole foot trajectory of PD patients shank as the disease progressed. However, the first principal component related to the forward displacements mainly contributes to distinguishing between mild and advanced PD patients (Figure 6D). In addition, in distinguishing between mild PD and advanced PD patients, the accuracy was higher when using the first two principal components than when using the six gait features (Table 2). Thus, the small forward displacements would characterize the gait of advanced PD patients compared to mild PD patients. However, the accuracy of distinguishing between the mild and advanced PD patients was lower than over 10% than between the mild PD patients and healthy older individuals. Therefore, small forward displacements would not characterize advanced PD patients compared to large vertical displacements in mild PD patients.

Many studies have sought to distinguish PD patients from healthy subjects using gait features and machine learning technology (Barth et al., 2011; Sant’Anna et al., 2011; Klucken et al., 2013; Cuzzolin et al., 2017; Micó-Amigo et al., 2017; Caramia et al., 2018). As movement impairment in PD patients increases with disease severity (Hausdorff 2009; Hass et al., 2012), a classification that includes advanced patients should be easier than that which includes only mild PD patients (Rovini et al., 2017). Klucken et al. (2013) extracted 694 temporal and frequency features from measurements during gait and toe-tapping tasks through IMUs attached to the heel and used linear discriminant analysis, adaptive boosting, and linear/nonlinear SVMs as classifiers. They found that the classification accuracy for mild and advanced PD patients and healthy older individuals reached only 82% and that it was mostly the mild PD patients that were misclassified. Therefore, temporal and frequency features may compromise the classification between mild PD patients and healthy older individuals when using few IMUs and simple gait tasks. In the future, it should be investigated whether gait trajectory features contribute to the classification of patients with PD.

This study’s limitations include that the advanced PD patients were not age-matched to the mild PD patients or healthy older individuals. The accuracy of distinguishing between mild and advanced PD patients was lower than between mild PD patients and healthy older individuals (Table 2), for reasons that are not clear. As age affects spatial gait features, the accuracy of distinguishing patients with different PD severity should be determined by age matching in future studies. In addition, the PD patients who participated in our experiment were in on-state. Thus, the trajectory features of the PD patients could be affected by the medication. In the future, the foot trajectory of PD patients who have not yet been treated should be investigated. Furthermore, our results cannot be used for the assessment or diagnosis of PD. For assessment or diagnosis usage, more data regarding foot trajectory should be collected for machine learning and validation and tests should be conducted. In addition, multi-class classification should be used in the future. Furthermore, other pathologies can cause the larger vertical trajectory in PD patients compared to healthy older individuals. Thus, the foot-trajectory data of other pathologies should be also collected and classified.

Overall, we found characteristics in foot trajectory during gait in mild and advanced PD patients by employing IMU measurements. The forward displacements of mild PD patients were smaller than healthy older individuals, which were smaller than healthy young individuals. The vertical displacements of mild PD patients and healthy older individuals were larger and smaller than healthy young individuals, respectively. These results showed that mild PD patients decreased the forward displacements in the foot trajectory through the effects of aging and PD but increased the vertical displacements regardless of decrease by aging. All forward and vertical displacements of advanced PD patients were smaller than the mild PD patients. Thus, foot trajectory shrunk during the progression of PD. The principal component analysis and SVM revealed that the components, which related to the vertical and forward displacements, mainly contributed to the accuracy of distinguishing between mild PD patients and healthy older individuals and between mild and advanced PD patients, respectively. Therefore, the vertical and forward displacements in foot trajectory characterized gait of mild and advanced PD patients, respectively.

## Data Availability

The datasets presented in this article are not readily available because the data set for this study is not publicly available because there is an agreement for data exchange between the Tokyo Institute of Technology and Kanto Central Hospital. Requests to access the datasets should be directed to TO, ogata@c.titech.ac.jp.
